# Pathology and Treatment of Yellow Fever; with Some Remarks upon the Nature of Its Cause and Its Prevention

**Published:** 1881-09

**Authors:** H. D. Schmidt

**Affiliations:** New Orleans, La.


### ARTICLE VII.

THE PATHOLOGY AND TREATMENT OF YELLOW FEVER; WITH SOME REMARKS UPON THE NATURE OF ITS CAUSE AND ITS PREVENTION. By H. D. Schmidt, M.D., New Orleans, La. (Continued from page 178, August No., 1881.)

### REMARKS ON THE PROBABLE NATURE OF THE INFECTIOUS POISON OF YELLOW FEVER.

In the preceding part of this treatise, I first conducted the reader to the bed-side of the yellow fever patient, in order to take a general view of the clinical symptoms, such as they generally present themselves to the physician during an epidemic. After the study of these phenomena, I conducted him to the dead-house, and pathological laboratory, where I showed him the pathological changes in the organs and tissues which had taken place during the course of the disease; and lastly, attempted to draw a sketch of the complex of pathological processes constituting the disease, by comparing the clinical phenomena with the pathological facts revealed by the autopsy and microscopical examination, the whole representing the general pathology of yellow fever.

In discussing the latter, I frequently referred to the influence or effect of the infectious poison, the original cause of the disease, but forbore to make any remarks upon its particular nature, which I shall now discuss. While in the preceding pages, however, we dealt with substantial, incontestable facts, with phenomena or things of a tangible nature, directly appreciable to our special senses, we now approach a subject which, on the contrary, is, to a certain extent, hypothetical in its nature, and where, unfortunately, too much space is left for the vagaries of the imagination. With the exception of cholera, there exists no disease of which the original cause or contagion has given birth to such a variety of speculations, both among physicians and laymen, as yellow fever. Inorganic and organic nature, the earth, the water, the atmosphere, minute plants and animals, the planets, and even invisible germs, have been suspected to be the cause. And accordingly, especially during an epidemic, when the daily newspapers are pregnant with yellow fever literature, there is no want of suggestions, and wise speculations, relating to that mysterious agent, the "germ" of the disease. In such times, high or low, wise or ignorant, everybody has an opinion, a view of his own on the subject, and without regard to whether it is based upon sense or nonsense. How cheaply feels the man of science and facts, the lover of truth and reason, in such a time. His voice is too weak to be heard in the turmoil, and all he can do is to observe and to collect, and to study the facts relating to the subject, to be conscientiously compared with those already established by other observers, at a time when the storm has passed, and the general excitement is worn out.

These remarks will not only show the difficulty, but, moreover, the delicate nature of the subject which I now propose to discuss; and if I fail in this task, it will not be owing to a lack of desire of presenting the subject fairly and without prejudice, but to my incapacity.

In the discussion of a subject, such as the probable nature of the infectious poison of yellow fever, of which the opinions of physicians differ so widely from each other, it is essential that the author should present it to the reader in all its various aspects, and be concise and definite in the terms of his demonstrations; while, on the part of the reader, in order to properly understand the arguments brought forward and to judge correctly, it is no less important that he should be familiar with the details of the subject. To comply with these propositions, it becomes necessary to introduce the subject with some general remarks.

As long as yellow fever has been known to the civilized world, a controversy has existed among medical men, especially among those of prominent seafaring nations, as to whether this disease is communicable from individual to individual, either in a direct or indirect manner, or whether its cause exists uniformly throughout the air of the infected district, having access to all persons alike. In other words, the question was, and still is—Whether yellow fever is a *contagious*, or a purely *infectious* disease.

As the terms "infection" and "contagion" have formerly been, and perhaps still are, by a number of physicians looked upon as synonymous, though they really convey different ideas, some explanation is demanded in what sense they will be used in this treatise. As far as I understand their proper meaning, the term "infection" signifies that the disease has been acquired by some noxious body taken into the blood from *without*, and without regard to whether the infectious poison is a product of the animal organism itself, or, on the contrary, a body *totally foreign* to it, which, whether *animate* or *inanimate*, owes its existence to external causes, whatever they may be. The term "contagion," on the other hand, implies that the *active poison emanated* from the diseased person or animal, and, if entering the blood of a healthy individual, gives rise to all the symptoms and organic changes by which the original disease is characterized.

Now, it is obvious that a purely *infectious* poison, as, for example, that causing miasmatic fever, hardly ever exists limited to one particular spot or center, but is rather distributed throughout the whole air of the infected district, and affects many persons at the same time; while a *contagious* poison, like that of small-pox, being *most probably* a product of the diseased organism itself, originally emanated from the affected person or animal, and extends its noxious influence by being inhaled or swallowed by other individuals in the vicinity. From this common center the disease spreads, either *directly* from individual to individual, or

*indirectly* by adhering to surrounding objects, such as clothes, bedding, furniture, goods, etc., upon which it may be carried to distant places, and, as in the first instance, propagate itself by entering the system of other individuals.

From this explanation it will be seen that some poisons may, in entering the organism, give rise to certain diseases not communicable from individual to individual, and be therefore purely *infectious;* while others, producing communicable diseases, and being *contagious*, are at the same time *infectious*, as they must necessarily enter the blood of the individual before they can exert their noxious influence upon the organism, and manifest their contagious nature. But notwithstanding this general trait of character, possessed alike by the *purely infectious* and *contagious infectious* poisons, the latter have a *special* trait besides, consisting in a certain permanency of the impression which they make upon the organism, and by which the latter acquires the so-called "*immunity*" from a second attack of the disease. Accordingly, a *purely* infectious disease may recur quite frequently, while the first attack of a *contagious* infectious disease protects the organism from another.

Although many causes have in the course of time been assigned to the origin of the various infectious diseases, nothing definite is as yet known about the intimate nature of their infectious poisons. The most prominent theories, at present entertained on the subject, are—the "gaseous," the "glandular," and the theory of the so-called "contagium vivum." The first refers the poison to gaseous or molecular substances, arising from the decomposition of vegetable and animal matters, or even from certain emanations from the ground of certain localities, known as *miasmatic* or *malarial* poisons. The second theory explains the poison as being of an animal origin, similar to the poison of snakes or other animals, or produced by certain morbid changes in the blood and tissues, called forth by its original introduction, and giving rise to morbid secretions possessing its specific properties. The theory of the "contagium vivum," lastly, refers the poison to certain minute organisms, which, entering the system through the lungs, alimentary canal, or even wounds, get into the blood, and give rise to the specific symptoms of the disease. Each of these theories, of course, exists in different minor forms or variations.

Now, as regards the cause of yellow fever, each of these theories, with its minor variations, has been put forward for its explanation, but without any palpable or positive proofs; for if anything regarding it can be proved, it is only negatively. With a considerable number of physicians, the theory of the contagium vivum, or so-called "germ or bacteria theory," being of a more recent date, has become especially popular. But as its origin and details are, as it appears, least understood of all others, I shall give it the preference for a full discussion, from which the reader may judge for himself whether it is applicable to yellow fever or not.

The theory of a "contagium vivum," relating to the cause of infectious diseases, is not of a recent date, but seems to have existed as a vague idea since the invention of the compound microscope, by means of which the existence of minute organisms first became known to the physician. At the same time another idea prevailed, according to which infectious diseases depended upon a certain process taking place in the blood, similar to that of alcoholic fermentation. These ideas assumed a more definite form by the discovery of *Schwann*, that the yeast-cell played an active part in this fermentation. Nevertheless, the germ theory, thus formed, lingered for a number of years, until it received a fresh impulse by *Pasteur's* proclamation, that every fermentative process was called into action by the agency of minute organisms performing the part of a ferment, and that each kind of fermentation depended upon a special kind of fungus. Pasteur's views, of course, were in opposition to the physico-chemical theory of *Liebig* then prevailing, according to which any organic matter, while in a state of decomposition, was able to induce molecular changes in another unstable, fermentable substance. Now, although the assertions of Pasteur, that all fermentations are accompanied by living organisms, have been generally admitted as a fact, the question, whether these organisms are the direct cause of the molecular changes, or whether they are to be regarded as epiphenomena, remains as yet undecided, and the theory of Liebig is, in a slightly modified form still upheld by a number of the most eminent chemists.

The doctrines of Pasteur were eagerly received by many physicians, and applied to the explanation of the cause of infectious and contagious dis[illegible]. The greatest impulse, however, which the germ theor[illegible]ed, was the discovery and description of minute organis[illegible]e blood of animals affected with malignant pustule or splenic [illegible], by *Pollender* and *Brauell*, in Germany, a subject which, several years later, was more closely studied by *Davaine* in France.

A still more definite form the germ theory received by the examinations of cholera-stools, made by *Klob* during the epidemic prevailing in Europe in the years 1865 and 1866. In examining, namely, the intestinal contents of cholera patients, he discovered a large number of very minute spherical bodies, together with others of a more oblong form, adhering to the outer surface of the epithelial cells. In the intestinal mucus, or in that of the cholera ejections, he also met with these bodies, collected in circumscribed patches in the form of so-called colonies, and imbedded into a gelatinous material. In certain parts of these colonies he observed a number of these bodies, liberated by the liquefaction of their gelatinous envelope, in active motion; while others, united in pairs, or adhering to each other in the form of longer or shorter rows, remained motionless. In the intestinal mucus he met with such colonies formed by oblong, rod-like bodies, either in pairs or, like the former, united into chains of different length.

From these observations, Klob drew the conclusion that all these bodies represented but a single organism in its different phases of metamorphosis, and by further inquiry found them, especially the rod-like form, to correspond with the *Zoglœa termo* of *Cohn*, or *Bacterium termo* of *Dujardin*. Meeting, however, with no other vegetable organism in the ejections, but always with large numbers of these bacteria, he concluded that these forms of fungi, constantly met with in the alimentary canal of cholera patients, never attained a higher development in this locality.

From this it will be seen that these bodies represented the same organisms always met with in stale urine, and other putrid substances, formerly known to physicians under the name of "vibrios," though the knowledge of their true nature and development, and the prominent part which they play in the process of fermentation, belongs to more recent times. They were already known to *Ehrenberg*, who regarded them as animals, and classed them with the *Infusoria;* but when their nature was rendered doubtful, *Haeckel* placed them, about thirteen years ago, in his newly established natural kingdom of *Protista*, composed of a number of other organisms of doubtful origin. *Naegeli*, a distinguished German botanist, formed them into a separate group of fungi, which he named *Schizomycetes.* In classing these organisms with the lower forms of fungi, however, a difficulty was encountered in the want of similarity regarding the precise mode of their reproduction, for while, with the exception of *Cryptococcus cerevisiæ*, or yeast plant, in all fungi certain typical organs of reproduction are known to exist, none could be demonstrated to exist in the schizomycetes. For, in the latter, the only mode of multiplication observed, consisted in a division of the minute rods in only one direction, while in the fungi, which are likewise represented by linearly arranged cells, the process of multiplication takes place by gemmation, not only in a straight line, but also by the formation of lateral branches. Finally, after having been studied and classified by other distinguished botanists, a second mode of reproduction, to be mentioned hereafter, was discovered in these organisms only a few years ago by artificial cultivation. In accordance with the importance they gained, their nature and their relations to organic substances were studied more closely, leading to the discovery of a large number of new species, and rendering the field they occupy so extended as to form at present a special branch of study in natural history. They have also been subjected to new classifications, especially by *Billroth* and *Cohn*, too extensive to be followed by the practicing physician. But, as in a few instances, particular forms of bacteria are found associated with some special pathological processes occurring within the animal body, and also with the different kinds of fermentation observed in fermentable substances, I deem it proper to give a short sketch of the lower fungi in general, and of those species of the schizomycetes in particular, which have hitherto been observed in association with these processes.

As regards the lower forms of fungi in general, *Prof. Naegeli*,*

---

**C. v. Naegeli. Die niederen Tilze* in ihren Beziehungen zu den Infections Krankheiten und der Gesundheitspflege. Muenchen, 1877.

of Munich, one of the first authorities in Botany, divides them in three natural groups, as follows :

1. *Moulds*, representing branched, segmented or unsegmented filaments.

2. *Sprouting fungi* (yeast-cells, saccharomyces, mycoderma, etc.), representing spherical or oval cells, multiplying by means of sprouts from their surfaces, and living either singly or united to form arborescent colonies.

3. *Cleft fungi* (schizomycetes, putrid-fermentation cells, micrococcus, bacterium, vibrio, spirillum, etc.), are spherical cells multiplying by division, and met with either singly or united into rows (rods, filaments), rarely into cubes. They represent the smallest organisms known, the weight of 30,000 millions of the smaller forms would scarcely amount to one milligram.

"The moulds slowly destroy organic substances by consuming them for their nutrition, while the sprouting and cleft fungi act besides as ferment in decomposing large quantities of matter by fermentation."

"The moulds and sprouting fungi are closely related, though, with the exception of one case in which the same fungus (mucor) appears in two forms of vegetation, they have as yet not been directly traced from one form to another. The cleft fungi, however, stand in no genetic relationship to the two other groups, as they can neither produce other forms of fungi, nor take their origin from these."

"The natural species of the lower fungi are not developed in such a manner as to have particular functions of decomposition corresponding with them. If there are different species existing among the cleft fungi, then each of them will cause different decompositions, as, on the other hand, the same decomposition may be caused by different species."

The following classification of the *Cleft fungi* or *Schizomycetes*, will suffice for all practical purposes.

The most simple form in which bacteria are met with is that of minute spherical or oval granules or cells, designated by *Cohn* the *sphæro-bacteria*. These are identical with the *micrococci* of *Hallier*, measuring from $\frac{1}{1000}$ to $\frac{1}{1200}$ mm. in diameter, and are often found aggregated into so-called "zogloea" or colonies, held together by a gelatinous intercellular substance, or, also, forming bead-like chains. They are motionless, and are sometimes found in putrefying substances in company with other bacteria, and and have also been considered identical with those granules found in the vaccine lymph.

The next group is represented by *bacterium termo* and *b. lineola.* The first presents the form of a minute rod or dumb-bell, has a slow, vacillating motion, and measures from $\frac{1}{360}$ to $\frac{1}{480}$ mm. in length; it is always found associated with putrefactive fermentation. The other is somewhat larger, and exhibits more rapid movements, being found, in company with b. termo and other forms, in stagnant water, as also in putrefying infusions.

The members of the third group are distinguished from the true bacteria by adhering to each other in the form of chains or "linked rods." It consists of *Bacillus*, representing a transversely lined filament, and of *Vibrio*, appearing as a cylindrical and curved filament. There are three species of *bacillus.* The first is *b. subtilis*, a slender thread, about $\frac{1}{20}$ mm. in length, and exhibiting a pausing motion. The second, *b. anthracis*, or *bacterium carbuncolare*, represents a motionless, oblong, and highly refractive body, found in the blood of animals affected with splenic fever, and measures from $\frac{1}{400}$ to $\frac{1}{80}$ mm. in length. The third is *b. ulna*, a stiff filament of $\frac{1}{26}$ mm. in length, and of greater thickness than *b. subtilis.* The *vibrios* are distinguished from the preceding forms by their rotary motion. The *v. regula* represents a flexible thread, from $\frac{1}{100}$ to $\frac{1}{48}$ mm. in length, distinguished by one or two slight curves and its slow motions. The other, *v. serpens*, measuring about $\frac{1}{80}$ mm. in length, is thinner than the preceding, and distinguished by the greater number and regularity of its curves and its more rapid serpentine motion.

The fourth group consists of the *spiro-bacteria*, distinguished from the vibrios by the greater regularity and closeness of the curves of the spiral, and their uniform, cork-screw motion. They are represented by *spirochaete*, to which the spirilla, found in the blood of relapsing fever patients, belong; and by *spirillum*, fine threads of a cork-screw form with a rapid spiral motion.

Nearly at the same time with Klob, *E. Hallier*, a professor of Botany in Jena, had also made a number of microscopical examinations of cholera stools, and for some time agitated the medical world by the discoveries he claimed to have made, consisting in certain spherical and oblong vesicles, filled with minute yellowish and glistening cells or spores, and also of certain spherical masses of minute roundish bodies, held together by a homogeneous gelatinous substance. To the granules or cells of these masses (colonies), adhering in large numbers to the remains of food and to epithelial cells, Hallier applied the name of "Micrococcus." Obtaining almost the same results from other examinations of cholera stools and vomited matters, sent to him from other places, he undertook, by certain methods, to cultivate the fungous elements they contained; and after the lapse of several days, he really found an increase in the forms of micrococcus, cryptococcus, torula, and leptothrix. In some of these cultures only certain forms of fungi appeared, while in others, other forms were observed, such as oïdium lactis, mucor racemosus, and also penicillium glaucum.

These examinations, made upon old material, could not have, as will be readily understood, any important bearing upon our knowledge of the cause of cholera, and for this reason could not but meet with much opposition and criticism on the part of other investigators. Nevertheless, Hallier drew from these observations the conclusion that those minute granules of micrococcus were able to give rise to various forms of fungi, and, moreover, that the particular form of a fungus depended upon the particular material in which it was cultivated. Thus, from each species of fungus micrococcus spores might be derived, which, according to the nourishment they received, would be developed into one or the other species of fungus.

Thus far, the whole question concerning the metamorphosis of one species of fungus into another would have remained botanical in its nature, if Hallier had not extended the bearing of his observations to the field of medical science in asserting that all infectious diseases were caused by the presence of micrococcus spores penetrating into the capillaries of the human organism, in which they multiplied, without ever being developed into a perfect form of fungus. Accordingly, each form of micrococcus would give rise to a particular disease. The same theory he applied to the process of fermentation, in which, according to the nature of the fermentable substance, a different fungus was produced. The application of Hallier's fungi theory to the causes of infectious diseases met with considerable opposition in Germany; and being finally rejected, made room for the so-called "bacteria-theory," which, though *apparently* supported by a few observed facts, to be discussed directly, is still very far from being established as a truth applicable to all infectious diseases.

Let us now take a glance at those diseases in which bacteria are almost constantly met with in the blood of the affected individuals. There are, in reality, only two in number—viz., splenic fever, and typhus recurrens, or relapsing fever,—to which may be added purulent septicæmia, and pneumo-enteritis of the pig, described by *Klein.* In the latter, however, *no bacteria are found in the blood of the diseased animals;* they can only be obtained from the inflammatory exudations by artificial cultivation. Besides, bacteria have *occasionally* been found in the blood of diphtheritic patients, and also in the vesicles of vaccina and variola. The first and last of these diseases are confined to our domestic animals, though splenic fever is also communicable to man.

In examining these diseases separately, we shall begin with *splenic fever* (malignant pustule, anthrax), the disease in which bacteria were first discovered in the blood of the affected individuals. It is chiefly met with in the sheep, cattle, hogs and horses, but may also be communicated by inoculation to smaller animals, as the rabbit, guinea-pig, mouse, etc., and even to man. It has frequently appeared in the form of an epidemic, spreading over certain districts, but is mostly confined to certain rural *localities,* even particular *pastures* or *stables.* The animals affected with the disease usually die one, two, or three days after the appearance of the first symptoms. One particular form of the disease, called the "apoplectic," is, however, observed, which proves fatal a few hours after its appearance. Death is accompanied by dyspnœa and cyanosis; and the chief anatomical changes revealed by the autopsy are enlargement of the spleen, a laky, viscid, dark-colored blood, numerous ecchymoses in different organs, especially in the heart, hæmorrhagic infiltrations into the areolar tissues, and also hæmorrhages into the alimentary canal. The particular phenomenon, however, now looked upon as pathognomonic of the disease, and bearing directly upon our subject, are the *bacteria* (bacillus anthracis) almost invariably found in the blood of the affected animals, in which they were first discovered and described by *Pollender*, more than twenty-five years ago, and the discovery corroborated soon after by *Brauell.* But it was not until several years afterward that the subject was taken up, and more closely investigated by *Davaine*, *Colin*, *Pasteur*, and, of late years, by very numerous other observers.

The statements of Davaine, that these bacteria represented the primary cause of splenic fever, and which first attracted the general attention to this subject, met for some years, however, with considerable opposition. Thus, when in 1869 the French Government appointed a commission, consisting of *Bouley*, *Teilhard*, *Lethérisse*, *Maret*, *Tournadre*, *Bonnet*, *Baillot*, *Chauveau*, *Richard*, *Felgéres*, *Messonier* and *Sansom*, to investigate the cause of this disease, Bouley, the chairman, reported as follows: 1. That the disease will be produced by inoculation, even with blood containing no bacteria; 2. That the anthrax blood, containing bacteria, loses its violent properties by dessication, without regaining them by the addition of water, though the bacteria may still be present; 3. The blood of rabbits dying from splenic fever always contained bacteria, whether the disease had been produced by inoculation with blood containing bacteria, or with blood containing some of these organisms. Finally, experiments made by inoculating calves and sheep, showed that bacteria were not always present in the blood, and that the blood proved equally virulent, whether containing bacteria or not.

Now although in the majority of cases of splenic fever bacteria are met with in the blood and tissues of the animals, there have been, nevertheless, a number of cases observed in which they could not be found, and others in which they could only be discovered shortly before or after death, a circumstance which gave rise to many doubts as to whether these organisms were really the primary cause of the disease, or whether they were to be regarded as epiphenomena. These exceptional cases, however, were explained, only a few years ago, by *Koch*, who, by artificially cultivating the bacteria in the anthrax-blood, discovered that the rod-like, motionless bacterium lengthened into a filament, in which finally a number of very minute bright spores appear. These, when liberated by a disintegration of the filament, develop into other rods, and when introduced into the blood of an animal, rapidly increase by transverse fission. From these observations it was supposed that in those fatal cases of splenic fever in which, in the beginning of the disease, no bacteria had been met with, the germs only had been present, to develop into rods shortly before death.

Notwithstanding this discovery, the question was still disputed, and gave rise to the most animated discussions in the medical-scientific world, and to very numerous investigations upon the subject, with hundreds of experiments upon various kinds of animals. Thus facts were brought forward from one side, to be disproved and contradicted from the other. But in carefully studying the results of the numerous investigations, the impartial observer must confess that in splenic fever, at least, it *appears* that the noxious influence upon the organism must be attributed to the presence of these bacteria. Perhaps the strongest proof of this assertion was forwarded, several years ago, by Davaine, consisting in the fact, afterward corroborated by others, that in pregnant animals the blood of the mother, containing bacteria, will, if introduced into the blood of a second but healthy animal, give rise to the same disease, and moreover cause certain death; while the blood of the fœtus is free from bacteria, and unable to reproduce the disease in another animal or to cause death. The placenta, therefore, performs in this case the part of a natural filter, allowing the liquor sanguinis to pass through it and enter the circulation of the fœtus, while the bacteria with the blood corpuscles are kept behind in the blood vessels of the mother.

Strong as this fact appears to be in favor of the correctness of the bacteria theory relating to splenic fever, it has not been sufficient to dispel all doubts on the question, which will probably not be satisfactorily solved until the origin of these particular bacteria is positively known,—that is, until it has been decided whether the affected animals receive them from the air they breathe, or from the water they drink, or whether they are contained in the ground of the infected pasture; and, lastly, whether they may not spontaneously be formed from organic granules, set free by the disintegration of some of the tissues within the animal itself, representing the process of "heterogenesis," as proposed by *Bastian*.

As regards their presence in the air, I have not met with any statement relating to it in the literature of splenic fever within my reach; though, regarding this matter, *Bender* suggested, a number of years ago, that these organisms might be contained in the green slime frequently covering not only the wooden troughs, but also the buckets and other wooden vessels containing the drinking-water of animals. *Bollinger*, who closely studied the epizoötic of splenic fever, prevailing some years ago among the domestic animals of the Bavarian Alps, states that in the Alps, as in all other places where splenic fever prevails, the condition of the ground plays a prominent part, inasmuch as the swampy and moist terrain of the Alpine pastures, like that of the valleys, favors the conservation and reproduction of the infectious poison, and that splenic fever never arises primarily from the ground, but is only produced when the latter has been previously impregnated with the poison, and that the so-called miasmatic origin of splenic fever cannot be proved. He thinks that the dung of the diseased animals plays a more important part than is generally imagined.

Davaine made some experiments for the purpose of showing that the splenic fever poison was transmitted from one animal to the other, and even to man, by the proboscis of flies; his statements, however, have been contradicted as well as confirmed by other observers.

Acknowledging the probability that in splenic fever the bacteria, themselves, represent the infectious poison, I forbear citing the numerous experiments, made by Pasteur and others, for the purpose of deciding the question, and the interesting discussions to which they gave rise, but dismiss the subject by reminding the reader of the fact, that these bacteria differ in some respects from those which have been met with in the other diseases mentioned; though *Ewart*,* one of the later investigators of the bacillus an-

* Quarterly Journal of Microscop. Science, April, 1878, p. 168.

thracis, states that micrococcus-forms, bacterium forms, bacillus-forms, and spore-bearing hyphæ are in nowise generally distinct, but that they are simply phases of the same life-history,—a life history doubtless common to all other bacteria.

The next disease to be considered is the *Typhus recurrens*, or so-called "relapsing fever," a contagious and epidemic disease, principally occurring in Germany, Russia and Ireland, but also in India; it is accompanied by affections of the spleen, liver, intestinal glands and kidneys, and by hæmorrhages in different organs. In the fever the pyrexia, lasting from two to six days, is followed by an apyrexia of eight or nine days; then follows a second paroxysm or relapse, lasting four or five days, and terminating in a profuse perspiration. Some cases terminate after the second paroxysm, though in many others other paroxysms are observed to occur. But the characteristic phenomenon of this disease consists in the appearance of minute filamentary organisms in the blood of the patient, first discovered several years ago by *Dr. O. Obermaier*, of Berlin. These organisms, called *spirochaete*, or *spirilla*, represent exceedingly fine and delicate filaments, from $\frac{1}{100}$ to $\frac{5}{100}$ mm. or more in length, appearing and moving very rapidly in the form of a close spiral, similar to a cork-screw. They are only observed in the blood shortly before and during the period of pyrexia, while during the apyrexia or intermission, they are entirely absent; neither can they be discovered in any of the secretions of the body. The singular relation existing between the appearance of these spirilla in the blood and the paroxysm of the fever, naturally suggests the idea that they probably are the true cause of the latter; and the explanation of the appearance and disappearance of these organisms in the blood of relapsing fever patients is, that while the spirilla die at the end of each paroxysm of fever, they leave their spores behind, from which during the apyrexial period, a new brood arises; these spores, however, have *as yet not been discovered.* As regards the transmission of the disease from one individual to the other by inoculation with the blood, the statements are contradictory, though it appears that it can be done, provided the blood is taken from the patient during the period of pyrexia. At the same time, however, the same results were obtained by inoculation with blood containing *no* spirilla, and taken from cases not differing from the others in severity.

In the July number, 1879, of the *Quarterly Journal of Microscopical Science*, an article, entitled, "The *Microphytes* which have been found in the *Blood*, and their *Relation* to *Disease*," by *Timothy Richards Lewis*, M.D., Surgeon, Army Medical Department, Fellow of the Calcutta University, was published, which originally formed part of the Memoir on the Microzoa and Microphytes of the Blood, appearing as an Appendix to the "Fourteenth Annual Report of the Sanitary Commissioners with the Government of India." This paper, in which Dr. Lewis, who is sufficiently known as an authority on this subject, discusses the whole germ-theory in an able manner, has attracted considerable attention; and since, as regards "relapsing fever," he speaks from personal experience and observation, I shall take the liberty of citing some of his remarks. He puts the question, why we should not at once admit that splenic disease is caused by bacteria rods, and that the aim of treatment should be the destruction of the vitality of these rods; or that recurrent fever is caused by screw-bacteria, and such remedial measures resorted to as tend to destroy them. Answering this question, he says:

"Before such views can serve as the basis of anything like rational treatment, it must be shown—1, either that these organisms, as ordinarily met with, are injurious when introduced into the animal economy; or, 2, that the forms found in disease are in some respects morphologically different from those known to be innocuous—such a difference, at least, as Virchow suggests, as exists between hemlock and parsley."

"With regard to the first point, it has been shown over and over again that all the representatives of the group of fission-fungi can be introduced into the system with the greatest impunity. Not only is their complete innocuousness practically put to the test by every individual at every meal, but observations have been published which have conclusively demonstrated that they may be introduced directly into the blood by injection into the veins, or indirectly, through the lymphatics in the subcutaneous tissue, without the slightest evil consequences. These facts are so well known and generally accepted that it is not necessary to refer to special observation."

"With regard to the second question, however, diametrically opposite opinions are held—all the advocates of the germ theory with very few exceptions, maintaining that the particular organism, in the particular disease in which they are specially interested, is wholly distinct from all others; that is, if the organism happens to be anything more definite than a granule or molecule. The diseases which have been specially cited as being associated with microphytes may be divided, roughly, into two classes, according to the form of the attendant microphyte — the septinous group, consisting of malignant pustule, septicæmia, and the malignant erysipelas or "typhoid" of the pig, on the one hand, and a low form of fever, commonly known as typhus recurrent, bilious remittent, etc., on the other."

"With reference to the organisms which have been found associated with the first-named group, taking malignant pustule as the type, it is to be observed that Mr. Robin in 1865 pronounced the bacteridia of Davaine to be identical with Leptothrix buccalis; and the well-known botanist Hoffman has stated his opinion that they do not differ from like bodies which appear in milk and in meat solutions. Ferdinand Cohn, again, in his observations as to the growth of bodies of the same character in hay solutions, declares that the bacilli in the latter are identical in form and size with those found in splenic disease, and that the various stages in their development correspond in every particular — the only difference which distinguishes them being that, whereas bacillus anthracis presented no movements, the bacillus of hay solutions did. This distinction, as has already been stated, has disappeared."

"Several years ago Dr. Cunningham and myself were, whilst conducting various observations together, frequently struck with the rapidity with which organisms appeared in the blood and tissues of animals after death in this country. The microphytes were not limited to minute spherical and elongated bacteria, but there were also present well marked staves and filaments. In a report submitted by us in 1872, and again in 1874, we drew attention to this matter and suggested the similarity between them and Davaine's bacteridia."

" A short time ago a circumstance occurred which drew my attention in a special manner to these organisms. Mr. Hart, a veterinary surgeon in Calcutta, forwarded to me for examination a little perfectly fresh blood which he had removed from a horse which had died that day of well marked anthracoid disease. His curiosity had been aroused as to the microscopical characters of the blood by perusing an account, in the *Veterinary Journal*, of ' worms' having been found in the blood of horses suffering from a similar affection in the Punjab. A slide was prepared and examined under the microscope at once, but no marked peculiarity could be detected ; but when this and other slides were re-examined twelve hours later, having in the meantime been kept under a bell-glass, numerous staves and filaments were observed, which, as to size and form, accurately corresponded with the description of like bodies characterizing the blood in anthracoid disease of Europe."

" Several cultivations were started by adding a little of the blood to fresh aqueous humor. The preparations were then set aside for a few hours in a moist chamber. As the temperature of the atmosphere at that time was generally over 90° F., no special appliances were necessary for supplying artificial heat. The development of the rods into filaments and subsequent appearance ot highly refracting oval bodies in the latter, corresponded so completely with what Cohn, Koch, Ewart and others had described, that it is not necessary to give figures of the changes that took place. A series of such cultivations was conducted by transferring a little of the last cultivation to fresh aqueous humor, and so on from one preparation to the other."

" It was then determined to ascertain whether the bacilli found in the blood of animals, which had been set aside for a few hours after death, would manifest, under like conditions, similar changes during their growth. Rats were obtained, killed by means of chloroform, and set aside for from three to twenty-four hours, or longer, according as the temperature of the atmosphere was high or low. The results proved that, almost invariably, bacilli were to be found in their blood, in the spleen and in other organs. On one occasion the rapid appearance of organisms after death was exemplified in a somewhat remarkable manner, and possibly the mode of death was not without some influence in determining their exceptionally early and plentiful appearance."

"The man employed to procure the rats, determined that he would get a sufficient number to last for some time, and proceeded to a large granary with his rat-traps. Having, however, found that he could procure more than could be accommodated in the cage which he had brought with him, he obtained a large earthen vessel, transferred twenty-seven rats into it, and tied a piece of cloth over the mouth of the vessel. As may be supposed, the rats had perished before he got home—all except one."

"I examined the blood and the spleen of twenty of these rats, within about six or eight hours after their having been caught, and found in each case that there were innumerable bacilli present, in every way morphologically identical with bacillus anthracis. In some of the cases the number was astonishing. They were present chiefly in the form of rods, but here and there some were seen to have grown to such a length as to cover two fields of the microscope."

"This experience tends to give support to the statement made by M. Signol before the French Academy, to the effect that motionless bacilli, identical with those found in carbon, will be found in sixteen hours or less after death in the blood of animals which have been asphyxiated by means of a charcoal fire. M. Signol, moreover, found that eighty drops of this blood would kill a sheep or goat very rapidly, notwithstanding that putridity could not be detected, so far as appearance and odor went; but that bacilli would not be found in the blood of the inoculated animals, either before or immediately after death."

"It has been urged that the microphytes which appear in the blood after death simply make their way into it from the intestinal canal as a result of the breaking down of the tissues. This objection is certainly no longer tenable, for many observers have shown that if some of the organs be removed from the body immediately after death, or indeed isolated from the circulation whilst the animal is still alive and under the influence of chloroform, these organisms will nevertheless appear if the preparation be kept for some hours at a suitable temperature."

In discussing the probabilities in favor of the bacilli and spirilla of the blood being epiphenomena, Dr. Lewis remarks:

"There is one circumstance in connection with the microscopic appearance which these organisms sometimes present, which deserves special mention, as it may serve as an explanation for their sudden disappearance from the blood; and that is, that they may present a well marked beaded or rosary-chain appearance. This feature I was able to observe on one occasion only. The spirilla of the ordinary character were plentiful in this person's blood on the evening previous to the day on which this observation was made, but when examined on the following morning, there were only linked or rosary-chain spirilla in his blood. They were not very numerous, and their movements were not of that *rushing* character ordinarily observed, but conveyed the impression of tumbling across the field."

"The inference which such an observation appears to warrant is, that when the blood acquires a certain as yet undetermined condition, it becomes unadapted to the existence of spirilla, and that the fibrils thereupon undergo segmentation, after the manner of other schizomycetes, and the separate plastides become diffused throughout the circulation; possibly, they then gradually disappear in the same manner as we have seen other plastides (minute bacteria, etc.) disappear after being injected into the circulation. This appears to me the more probable than that they continue in the circulation until the blood re-acquires the state suitable to their growth into fibrils, seeing that the time for their return is so uncertain—it may be two days, may be six days, or a fortnight even, and perhaps they may not return at all. Be that as it may, it is clearly evident that their existence as spirilla is dependent on the composition of the fluids of the body."

"Heydenreich suggests that their disappearance is due to the elevated temperature of the blood at the height of the paroxysm. If that were the case, they ought to become more numerous with the fall of the temperature after death, but it is well known that they disappear exceedingly rapidly when life becomes extinct, in this respect offering a marked contrast to other members of the cleft-fungi group—bacteria and bacilli."

"The fact of their total disappearance immediately after death, probably even before death actually taking place, is very significant, as showing the extremely close relation which exists between them and the blood in *living* tissues, seeing that when the blood is removed from the body, the spirilla will, under favorable conditions, retain their power of locomotion for several hours or days. What these subtle changes of the blood during fever processes may be, chemistry and physiology have not yet revealed; we can therefore only judge of them by the changes of temperature, etc., of the patient; and, in the particular condition under consideration, by the occasional appearance and re-appearance of spirilla, whose presence is manifestly dependent on antecedent changes. That the temperature commences to rise, and that other subjective symptoms are manifested before the appearance of spirilla, testifies to this, for it cannot be that they can exert an influence before they are themselves existent."

"Dr. Charles Murchinson, at the discussion on the germ-theory of disease at the Pathological Society, put this matter very closely when he said: 'The fact that in relapsing fever and sheep-pox distinct forms of bacteria have been found, in no way proves any casual relationship between these diseases and the bacteria, and is readily accounted for by the acknowledged fact that the form taken by many minute growths depends not upon the germ, but upon the nature of the medium in which it grows. Indeed, the observations which have been made on the spirilla of relapsing fever are strongly in favor of this view, for they are present in the blood during the first paroxysm, but disappear before the crisis; are absent during the intermission, but return with the relapse of the fever, and again disappear before the crisis. It seems difficult to account for their appearance and annihilation twice over, except on the supposition that the soil was suitable for their development during the febrile process, and unsuitable when the febrile process was complete.' A like conclusion must be arrived at regarding the bacilli in malignant pustule, septicæmia, and the so-called typhoid fever in the pig, horse and other animals. With regard to the microphytes just named, it may be confidently stated that they are never to be detected in the earlier stages of the disease, but only at a brief period before and after a fatal termination. To my knowledge they have never been found in the blood of animals which have subsequently recovered; they have always been recognized only as one of the concomitants of impending dissolution."

The next disease in which bacteria have been met with in the blood is *Septicæmia*, representing a depraved condition of the system, and generally observed in connection with grave surgical injuries, such as extensive wounds, etc., or following some operations, a condition characterized by all the symptoms of blood-poisoning. The anatomical changes observed after death are as follows: Early decomposition, skin of a dirty yellowish tint, with numerous livid spots, due to local congestion. Blood of a dark color, fluid, or imperfectly coagulated; small extravasations found in the heart. The lining membrane of the heart and aorta is more or less stained by imbibition of the coloring matter of the blood. The spleen is usually large and soft, very friable and pulpy; the parenchyma of the liver and kidneys is congested or undergoing more or less granular degeneration. In a number of cases, metastatic abscesses are found, especially in the lungs and liver. In surgery, septicæmia is identical with pyæmia, a condition which for a long time was supposed to depend upon the absorption of pus from the surface of the wound into the blood. Though it remains doubtful whether healthy pus could produce this condition, it appears to be generally accepted that the infectious poison enters by the way of the wound, to give rise to a putrid infection of the whole organism, accompanied with or without the formation of metastatic abscesses. But, as in the latter, which are supposed to be formed by embolism, bacteria were found in many cases, this fact was taken hold of by the germ-theorists, and the question arose, whether the septicæmic phenomena were due to the absorption into the blood of a specific septic poison, arising from putrid changes in the pus secreted from the granulating surface of the wound, or even to a putrid decomposition of certain emboli in the blood vessels; or to the noxious influence of *bacteria* contained in the surrounding air and finding their way into the blood through the surface of the wound. The antiseptic dressing of *Lister* is based upon the assumption that the bacteria, settling upon the wound, cause a putrefactive fermentation in its secretion, which, if absorbed into the blood, gives rise to the symptoms characterizing septicæmia.

The idea that septicæmia depended upon the presence of a putrid poison in the blood gave rise to very numerous experiments made upon animals for the purpose of artificially producing this condition by the inoculation or injection of various putrid substances, such as putrefying blood, muscle, urine, etc., containing numerous bacteria, and, as was expected, resulted in the production of the same or similar symptoms and pathological changes in these animals, as characterize septicæmia. While some of these animals died, others remained unaffected. When, however, the blood of these animals, *containing bacteria,* was inoculated into others, graver symptoms, terminating in death, were produced, showing that the putrid poison had increased in violence, but without *being accompanied by bacteria.* Davaine, who extended this successive inoculation upon a very large number of animals, found that the intensity of the poison was increased with each animal through which it passed, and that thus the one trillionth part of a drop would suffice to kill a rabbit. The latter presumption, however, has been refuted by Chassaignac, Onimus, and others. The pathological changes found in animals killed with septicæmic blood are similar to those of septicæmia in man. They are: peritonitis, pleuritis, enlargement of the spleen, pneumonia, hyperæmia of the kidneys, jaundice, and hyperæmia of the intestines. But while bacteria are almost always found shortly before or after death in the blood of animals inoculated with anthracoid blood, it is only an exception to the rule when they are met with in the blood of animals inoculated with septicæmic blood. In fact, all symptoms of septicæmia may be observed, *without the appearance of bacteria in the blood.* The bacteria found in septicæmic blood or abscesses mostly belong to the first group, the sphero-bacteria, and are generally met with in the form of zoglœa-patches. In fact, with the exception of splenic and relapsing fever, it is almost exclusively the micrococcus form that has been met with in other diseases, wherever bacteria have been met with in the blood and secretions, or upon suppurating surfaces. And it is especially this fact which the germ-theorists have hitherto failed to bring into harmony with their theory.

As in splenic and typhus recurrent fever, in septicæmia, also, the question has been whether these bacteria, found in a number of cases, are the direct cause of the disease, or mere accidentally accompanying phenomena ; or, whether the symptoms of the disease depend upon the influence of a noxious poison formed within the organism itself. In order to decide this question, various experiments have been made, too numerous to be cited in this place. In these experiments, the most difficult part was to effect a separation of the bacteria from the putrid liquid containing them, and different methods have been followed to accomplish this task, one of which, first resorted to by Pasteur, consisted in filtering the liquid through porous substances, like burnt-clay vessels, by which proceeding the bacteria were left behind in the residue upon the filter. Separate inoculations with the filtered liquid or with the residue, were then made upon different animals, but as the statements of the results of these experiments are so contradictory, I forbear mentioning them ; for while, on the one hand (Pasteur) it is stated that the filtered liquid had lost its virulence, we are told by other observers (Anders, Satherthwaite, Curtis and others) that the septic poison, after the destruction of the bacteria, retains its noxious properties. Even injections of putrid liquids, containing bacteria, into the blood of animals, is not always followed by septicæmic symptoms. Thus *Livon*, who injected such liquids into dogs, observed no traces of parasites in the blood, nor any pathological changes in the organs. Davaine found that septicæmic blood loses its virulence by putrefaction, and, moreover, that the blood taken from *living septicœmic* animals had no deleterious effects upon the animals when introduced into the blood.

The micrococcus form of bacteria, besides being met with in the blood and secretions of pyæmic patients, has in a limited number of cases also been observed in diphtheria, especially in the diphtheritic exudate and ulcers, through which, in some instances, they may have found access to the blood; furthermore, in the vesicles or blisters of erysipelas, and even, in exceptional cases, upon the valves of the heart in endocarditis. The experiments and observations relating to the presence and signification of bacteria in the diseases mentioned are very numerous, and the literature of the subject has become too extensive to be thoroughly discussed in this place. Nevertheless, in order to enable the reader to judge for himself, I shall cite the observations and views entertained by some of the leading pathologists. Thus, *Billroth** frequently found cocco-bacteria in the different normal fluids of the body, especially in the serum of the pericardium; but by no means constantly in the blood of the cadaver of persons who had died of septicæmic diseases, even not in the purulent contents of abscesses, etc. The report is as follows:

"The essential cause of their accumulation was most probably here and there the higher or lesser degree of decomposition of the parts. A conditioning relationship of these organisms to the coagulation of the milk, as well as to the alkaline fermentation of the urine cannot be presumed, as they can be demonstrated, here and there, long before the commencement of the process. It is known that the pus of ordinary, apparently healthy granulating wounds contains numerous cocco-bacteria; also, that the quantity of those present stands in no definite proportion to the height of the fever or other general phenomena of the patient. In the reverse, cocco-bacteria are found in the pus of closed abscesses without the co-existence of fever, while, on the other hand, in similar cases, high fever may exist without cocco-bacteria being detected in the pus. Finally, the presence of cocco-bacteria cannot arbitrarily be asserted from the bad odor of the pus. A very similar relation exists in erysipelas; while, namely, the serous contents of the blister in half of the cases examined contained no cocco-bacteria, they were found several times under the epidermis in non-specific exudations."

"All forms of cocco-bacteria may be produced by artificial cultivation from those parasitic forms found in the organism under different pathological conditions, though none of them would, in its different phases of development, show any essential character differing from those bacteria associated with putrefaction. From this it results that not even a specific mode of appearance is observed on the bacteria met with in the animal organism, opposite to those found in inorganic nature. Still less can this be asserted for definite pathological conditions, or for individual diseases."

---

**Virchow u. Hirsch*, Jahresbericht f. d. Jahr 1874. Vol. I, p. 353.

"The conditions essential to the regular growths of the cocco-bacteria are only partially known and certainly very complicated; the presence of decomposable organic substances is by no means sufficient, which is proved by the want of correspondence between the commencement of putrefaction and the appearance of these organisms, and also by a want of a definite proportion of the quantitative extent of the latter to the putrefaction. The presence of water and air, as well as a certain passivity and undisturbed position of those parts serving as a place for their germination and proliferation, are undoubtedly essential for the development of the organisms. But even then, a further increase in numbers does not take place, as the experiments of Max Wolff have proved, who failed to observe any septic phenomena after the introduction of fungous spores into the trachea, though he could detect the particles in question in the circulating blood. The numerous statements concerning the presence of bacteria in the blood in this or that disease, also, must be received with great care, as there are other very pronounced cases of septic disease in which they are, at least in entirely fresh blood, certainly wanting. It appears, therefore, that the 'vitality' of the organism exerts a certain conditioning influence upon the destruction, that is, the further growth of the accumulated fungous germs in the circulating blood, as in the secretions and exudations, etc. This fact may be better understood in presuming that the cocco-bacteria are unable to assimilate the albuminous bodies of the tissues in the form in which they exist in the living organism."

"In order that this assimilation may take place, a third hitherto unknown body, which Billroth designates as 'phogistic zymoïd,' must take part, that is, a body which, produced in the course of the inflammation and by this process itself, excites the fluids of the tissues, the blood, pus, etc., in the way of a ferment, rendering them a suitable and accessible medium for the multiplication of the dormant germs contained in it. This phlogistic zymoid acts wherever it comes in contact with healthy tissues, but still more rapidly upon diseased tissues, in exciting them to inflammation and causing their destruction. By the disintegration of the tissues thus induced, the ground becomes prepared for the unlimited proliferation of these parasitic vegetations. In speaking of the putrid infection, Billroth declares it very probable that the respective poison arrives in the organism in the form of a finished chemical body, exhibiting its effects in proportion to its respective quantity : a conception standing in opposition to the generally adopted fermentation theory."

"*Panum*,* in continuation of his former investigations concerning the relations of the parasitic organisms to putrid processes, published in 1856, renews the question whether the microscopical organisms contained in the ordinary putrefying liquids, can be regarded as the intermediate cause of that group of symptoms designated putrid or septic infection. As he was unable to deprive, by long continued and energetic boiling, putrid liquids of their noxious properties, by which the vegetable organisms contained therein must necessarily be killed,—he arrived at that time at the conclusion that their deleterious properties could not be ascribed to the micrococci and bacteria, but to another lifeless agent, representing a pure chemical body. Before passing to his more recent experiments, corroborating this view, he endeavors to demonstrate by deduction the improbability of the doctrine, according to which the bacteria are the direct septically acting agents. He mentions the fact, confirmed by Magendie, Gaspard and Stich, that the intestinal canal of healthy men and animals contains an extractible substance, which injected into the blood gives rise to all phenomena of sepsis. The circumstance that the latter do not appear under normal conditions, has been made use of as a proof for the molecular, insoluble nature of the poison. But bodily substances also, even psorosperm-eggs, may pass through the epithelium of the intestine, while many soluble substances, as a solution of curare, for example, pass with extraordinary difficulty. Against this supposition is, furthermore, the fact that the duration of the time of incubation in septic diseases does not stand in the reverse proportion to the quantity of the injected liquid, which certainly should be the case. As regards, finally, the clinical symptoms of the septic infection, Panum finds the characteristic points of the course not in the curve of temperature, but in the affection of the intestine, announced by vomiting and

* L, c. p. 354.

diarrhœa ; then in the severe collapse and depression of the entire nervous system, and finally in the rapid appearance of putrefaction and the imperfectly coagulating blood. From this it is clear that we are not justified in forming from the symptoms of the poison, and especially from the duration of the course of the affection depending upon the dose and the concentration of the injected liquid, an argument for the essential participation of the at the same time introduced microscopical organisms."

"Against the bacteria-theory, the fact that a putrefying liquid, rendered perfectly free and clear from bacteria by repeated and continued filtration, loses nothing of its septic effects, may be most strikingly brought forward. Of the entire absence of bacteria, Panum became convinced by means of high amplifications. By a repetition of the experiments with putrid liquids, which had been boiled for several hours, he became still more convinced that the distillate obtained from it, though of a bad odor, was not poisonous, while the deleterious effect of the residue was little less than that of the original liquid. The alcoholic extract of the clear filtrate of meat, which had been macerated for four weeks, injected into the blood of a dog, shows only a hypnotic effect. The residue, not extracted by alcohol, however, gives rise to vomiting, inclination to stool, tenesmus, and general depression, from which the animals scarcely recover after the lapse of twenty-four hours. In view of such results, Panum regards it as settled that the putrid poison is no simple extractive substance, but a real chemical body; the latter being the true deleterious agent in the septic processes, while the part of the bacteria is only accidental."

"As regards the origin of this putrid poison, Panum suspects that it is produced by the vital process of the bacteria, perhaps a kind of secretory product (Bergmann). All experience relating to the presence of bacteria in the healthy organism of man, as well as in the diseased, teach us at least that, both bacteria and processes of putrefaction, stand in near relationship to each other. Though, undoubtedly, bacteria also appear in the body with decomposition, their increase always depends upon the cessation of life, be it from local or general causes."

"*Hiller*,* in concert with the results of his former researches,

* L. c., p. 365.

states that in putrefying urine the degree of development of bacteria does not always correspond with the height of the decomposition, but that both rather stand to each other in a reversed proportion. Thus, urine left in an open, or corked, perfectly clean bottle, does not, even after two months, undergo putrefaction; the latter, however, took place in some bottles closed with cotton, and even earlier. Notwithstanding this difference in the results of the experiments, all specimens of urine corresponded in containing a rich vegetation of bacteria after the lapse of only a few days. But this vegetation only attained a certain low degree, and then remained stationary, if afterward a putrid decomposition was not induced. As long as the urine standing open was acid, mycelii of Penicillium glaucum and Torula cerivisiæ would grow, but with the commencement of alkaline reaction, the former disappeared entirely, and the latter partially. As regards the influence exerted by various chemical agents upon the putrefaction, ammonia and carbonate of ammonia remained negative. Phosphate of potassa and soda, as well as tartrate of ammonia, promoted in a high degree the development of bacteria, without producing putrefaction. Specimens of fresh urine placed in a close vicinity to putrefying urine showed extensive masses of bacteria without putrefaction."

"The putrefaction of urine, therefore, cannot be looked upon as a physiological phenomenon of exchange of matter through the bacteria. The vegetation of the latter depends solely upon the supply of assimilable nutritive matter, which, being scanty in fresh urine, is only furnished in sufficient quantity through the putrefaction. As, now, the bacteria are powerless to split the complicated (nitrogenous) combinations in the urine, it requires, in order to nourish them, an agent that induces the decomposition. This agent becomes active through and with the putrefaction, and from this the coincidence, regarding time and extent, of putrefaction and bacteria may be explained.

"In setting aside the decompositions taking place in the urine, experiments with chicken eggs show moreover that bacteria alone are incapable of inducing a putrefactive decomposition in the albumen. From this Hiller concludes the untenability of the vitalistic theory; he is moreover of opinion that the ferments of putrefaction are represented by most minute proteïn-particles, which, being themselves in a state of putrefaction, are carried through the air to the substratum, to involve it in a similar decomposition."

Although it had been stated by Pasteur, and corroborated by other observers, that no microscopic organisms could be detected in the healthy blood, experiments were nevertheless also made to prove the contrary. Thus, *Fiegel* took pieces of different organs of freshly killed animals, and fastening them to a previously boiled silken thread, plunged them successively into boiling paraffine, until they were covered by a thick layer of this material. Though it was supposed that the boiling heat of the paraffine had destroyed every living germ at the surface of the pieces, and that the layer of paraffine covering them had prevented the access of fresh germs, bacteria were nevertheless found in the interior of the pieces after the lapse of several days.

These experiments, while attracting much attention, were repeated by other investigators; and as the results obtained corroborated the statements of Fiegel, it was concluded that the bacteria themselves, being always present in the blood, were unable to exert any influence upon the production of pathological processes. *Burdon-Sanderson*, also, investigated this matter, by repeating Fiegel's experiments with a slight modification, rather improving the process; and in referring to the experiment in one of his lectures on "The Infective Process of Disease," delivered in the University of London, about two years ago, he says that under the conditions described it seems to him quite impossible to suppose, either that germs could penetrate to the organ (the liver or kidney) from the outside, or that any germ encountered by the organ in its transference from the body of the animal to the basin could escape destruction. If, therefore, bacteria be found, they or their germs must have been there before the organ was plunged into the hot liquid. From these experiments, he then concludes that germs continually enter the blood by the way of the portal circulation, and as no bacteria exist in the circulating blood, the alternatives are that, either the bacteria are formed and immediately afterwards destroyed, or they are not allowed to germinate, because the conditions under which they are placed in the living organism are such as to prevent their development.

Burdon-Sanderson then fully endorses the correctness of Fiegel's statements, and moreover explains the phenomenon according to his own views. But in looking again at the other side of the question, it will be found that *Chiene* and *Ewart* definitely proved the absence of bacteria or any other organism, if removed and examined under a spray of a solution of carbolic acid, together with other precautionary measures, taken during the experiment to prevent the access of these organisms. From these experiments we may learn to receive the statements regarding the presence of bacteria in the living blood or tissues with a great deal of caution.

The last disease in which bacteria have been obtained by artificial cultivation from the secretions of the animals, is *infectious pneumo-enteritis* of the pig, as it has been termed by **Klein*, who investigated it two years ago. The anatomical changes met with in this disease are similar to those already mentioned in connection with splenic fever, consisting principally of lobular pneumonia, ulceration of the mucous membrane of the intestines, the liver, spleen, etc. The results of these experiments showed: 1. That the fresh blood of diseased animals does not, as a rule, contain the virus, as it fails to produce the disease when introduced into a healthy animal. 2. That the fluid as well as the solid lymph of the diseased peritoneum contains the virus in a very active state. 3. That parts of the diseased lung, ulcerated intestines, and diseased spleen contain the virus in a very active state; a frothy, blood-containing matter, found in the trachea and bronchi, also possesses infectious properties, showing that the breath of the diseased animal is charged with poison. 4. That infection is produced by cohabitation with a diseased animal, or by keeping healthy animals in a place whence a diseased animal has been removed.

By some other experiments, Klein furthermore showed that the virus may be cultivated artificially outside the body of an animal, as had been successfully done by Koch, in splenic fever.

The cultivation of the virus consisted in taking a minute portion of the lymph, and inoculating a drop of fresh aqueous humor

---

* *Quarterly Journ. of Microscop. Science*, April, 1878.

of a rabbit, which, being placed upon a glass-slip and arranged in such a manner as to prevent the access of air, was kept in the incubator for twenty-four hours at a temperature of 33° to 34° C. With a minute portion of this fluid another drop of aqueous humor was inoculated and treated in the same manner; and from the latter a third, representing the third generation. Two animals inoculated with this matter were affected with the disease.

In a similar manner the virus was cultivated from a minute portion of solid lymph of the peritoneum of a diseased animal, and the cultivation carried to a fourth generation. Two animals, having been inoculated with this matter, became smitten with the disease. And, in carrying the cultivation of the virus to the eighth generation, the matter remained still effective.

The microscopical examination of the cultivated liquids showed that they were the seat of the growth and development of a kind of bacterium, resembling Bacillus subtilis of Cohn, and representing a fine delicate rod, thinner than the Bacillus anthracis of Koch.

The interpretation which Klein gives of the results of his experiments appears to me, for several reasons, not calculated to inspire much confidence. Thus, failing in transferring the disease from one animal to another by the inoculation of the blood, he succeeded with the inoculation of the exuded lymph, showing that the virus really existed in the latter. In this paper he omitted to mention whether he had examined the blood and lymph microscopically, and whether, or not, he had detected bacteria in one or the other; but, as he resorted to artificial cultivation, it may be presumed that he did not. Obtaining at last these organisms with their spores, he looks upon the latter as the true cause of the disease, without explaining whence they came. If they had been the true cause of the disease, they must have originally existed in the blood, from which they might have gotten into the secretions, and Klein should have detected them in this fluid as readily as he discerned them in the cultivated liquid. Besides this, he says: "After many failures—owing to the introduction of *Bacterium termo*—I succeeded at last in obtaining, already in the second generation of original virus, a pure crop of bacillus and its spores." Now, as will be seen, that with the bacillus he also obtained bacterium termo, rendering it very probable that both were derived from the same quarter, that is, from the outside; or, if not, that both, according to Ewart, represented different phases of one and the same organism, and, perhaps, originated in the same manner, as the bacilli in the dead mice and rats of Dr. Timothy Lewis. At any rate Klein did not consider it *impossible* for other organisms to make their appearance in the cultivated liquid, as further on he remarks that "no other organisms appeared in these culvations," *i. e.*, after the bacillus had displaced the bacterium termo. As regards pneumo-enteritis of the pig, therefore, it is more than probable that the peritoneal exudate itself represents the infectious poison.

In comparing the four diseases above described with each other, we cannot but notice the similarity of their accompanying symptoms, and of the anatomical changes observed after death. They are all characterized by an affection of the spleen, lungs, heart and intestines, accompanied by hæmorrhages, ecchymoses, and metastatic abcesses, symptoms depending more or less upon a depraved condition of the blood, and resembling those of typhus fever. Therefore, it may be presumed that the presence of living organisms, found in the blood of the affected men and animals during the last stage of these diseases, is due to the diseased condition of this fluid, affording a favorable medium for their development, and that the diseases themselves are due to the introduction of some specific poison into the blood, representing a product of the diseased organism itself.

That organized bodies may exist in the blood of certain animals without interfering with the normal functions of this fluid is sufficiently proved by the discovery of E. Ray Lankester, in 1871, of a large infusorium in the blood of the common frog of Europe, *Rana esculanta*, which he called *Undulina ranarum;* and, furthermore, by my own, made almost at the same time, of a similar infusorium in the blood of our tree-frog during three successive years. Though these infusoria were quite numerous in the blood of these animals, nothing abnormal could be discovered in the blood of the latter, a considerable number of which I kept under observations for many months.

In addition to the experiments and opinions of a number of distinguished pathologists, which, in the preceding pages, I have brought before the reader, I shall, before dismissing the subject, cite some portions of a remarkable address, delivered, in 1875, to the Pathological Society of London, by Prof. H. Charlton Bastian,* the well known advocate of the theory of "Spontaneous Generation," whose persevering studies into the nature of minute living organisms have not been surpassed by any other investigator. In the course of this address he says:

"I will, however, now briefly enumerate the evidence which seems to me quite sufficient to disprove the probability of the existence of any casual relationship between the lower organisms and the diseases cited at the head of this section, and to establish, on the other hand, the position that the bacteria met with in diseased fluids and tissues are for the most part actual pathological products—that they are, in fact, engendered within the body, or are descendants of organisms owning such an origin, rather than of previously existing organisms introduced from without. It would take too long were I to enter upon a consideration of this evidence. I must therefore content myself with briefly summarizing the principal facts and arguments on which a judgment may be founded."

1. "The experiments of many investigators prove that the alleged causes of diseases may be actually introduced into the blood-vessels of lower animals by thousands without producing any deleterious effects in a large proportion of the cases."

2. "Bacteria, if not actually to be found within the blood-vessels of healthy persons, do nevertheless habitually exist in so many parts of the body in every human being, and in so many of the lower animals, as to make it almost inconceivable that these organisms can be the cause of disease. In support of this statement I have only to say, that even in healthy persons they may be found in myriads in and about the epithelium of the whole alimentary tract from mouth to anus; they exist throughout the air-passages, and may be found in mucus coming from

* The Microscopic Germ-Theory of Disease; being a Discussion of the Relation of Bacteria and Allied Organisms to Virulent Inflammations and Specific Contagious Fevers.—*Monthly Microscop. Journal*, August, 1875.

the nasal cavities, as well as in that from minute bronchi. They exist abundantly amongst the epithelial débris within the ducts of the skin, not only in the face, but in the other parts of the body. Fresh legions of them are also being introduced into the alimentary canal with almost every meal that is taken, whence they may, perhaps, readily find their way into the mesenteric glands, if not farther within the system. And, lastly, in persons with open wounds, bacteria are constantly to be found in contact with such surfaces, especially if the wounds are not well cared for, though the injured person does not necessarily suffer at all in general health."

3. "It is no answer to these difficulties to say that there are distinct species among the lower organisms, some of which are harmless, though others are poisonous (or so-called germs of disease). In support of such an opinion nothing can be alleged save some of the facts whose cause is doubtful; whilst again, such an interpretation may be brought the experiments of several investigators, showing that bacteria are the creatures of circumstance, and modifiable to an extraordinary degree. The last position is even admitted by Professors Sanderson and Lister. The former acknowledges that they are 'the lowest organisms,' and that they are 'much more under the influence of the conditions under which they originate and are developed, than organisms of any other class;' while Professor Lister's own work has compelled him to make an admission which, in the face of facts previously stated concerning the wide distribution of bacteria within the body, seems fatal to a consistent belief in the germ-theory of disease. He says: 'If the same bacterium may, as a result of varied circumstances, produce in one and the same medium fermentative changes differing so widely from each other, as the formation of lactic acid and that of black pigment in milk, it becomes readily conceivable that the same organism which, under ordinary circumstances may be comparatively harmless, may at other times generate products poisonous to the human economy.'"

4. "The considerations now to be mentioned suffice, in my opinion, to complete the discomfiture of the germ-theory as an explanation of the mode of causation of the disease with which we are at present concerned. It is this. It has been shown, on the one hand, that the virulence of certain contagious mixtures diminishes in direct proportion to the increase of bacteria therein; and on the other hand, it has been equally proved that fresh and actively contagious menstrua lose scarcely any of their contagious or poisonous properties after they have been subjected for a few minutes, when in the moist state, to a temperature which no living units can be shown to survive (212° F.), or after they have been exposed to the influence of boiling alcohol, which is well known to be equally destructive to all recognized forms of living matter. Such facts have been substantiated by Messrs. Lewis and Cunningham, Sanders and others."

"Having said this much in opposition to the germ-theory, let me as briefly enumerate the facts and arguments which seem to me to show the real relations of bacteria and their allies to the diseases in question. I turn therefore to the construction of an opposite doctrine."

"Admitting, in part, the very frequent presence of bacteria in diseased fluids and tissues, I consider that their presence and import should be differently explained. I say I admit the association in part, though I by no means admit it to the extent alleged. Bacteria are not, for instance, to be found in the blood of persons suffering from pyæmia, as might be inferred from former statements of Dr. Sanderson, which I have already quoted. My own experience in this matter seems to be entirely in accordance with that of Professors Billroth and Stricker. Neither do I believe that the presence of bacteria in inflammatory fluids has the significance which Dr. Sanderson attaches to it, since it has been ascertained by myself and others that the exudation fluid of sick persons suffering from disease of a totally different type are often similarly crowded with these lowest organisms, whilst the recent observations of M. Bergeron seem to show that they may be found even in freshly extracted pus from ordinary abscesses occurring in elderly persons."

"Now, it would seem quite obvious that the consistent advocate of a germ-theory of a disease can only successfully maintain such a doctrine if he can show, amongst other things, that bacteria are more capable of altering the character and chemical constitution of fluids of the body than they are themselves prone to be altered by independently instrated changes taking place in such fluids. It seems, therefore, like unintentionally cutting himself free from the theory to which he has hitherto adhered, when we find Professor Lister, in speaking of the assumed 'special virus of hospital gangrene,' going on to say that 'it is not essential to assume the existence of a special virus at all, but that organisms common to all the sores in the ward may, for aught we know, assume specific properties in the discharges long putrefying under the dressings.' This passage has a similar import to that of a quotation previously made. In both a first plea is assigned to the modifying influence of altered fluids; and however much the correctness of such supposition wonld tell in favor of cleanliness, free exposure, or even of antiseptic dressings, it is none the less inimical to a consistent holding of the theory on which Prof. Lister has chosen to base his system of treatment."

"But though such statements are adverse to the holding of a germ-theory in the only form in which it may be at all tenable, they are entirely in accordance with my own observations and views. I maintain, in short, that even the very existence of organisms in the fluids and tissues of diseased persons is for the most part referable to the fact that certain changes have previously taken place (by deviation from healthy nutrition) in the constitution and vitality of such fluids and tissues, and that bacteria and allied organisms have appeared therein as pathologicel products—either by heterogenesis, or by what I have termed archebiosis, or birth direct from a fluid."

"The evidence on which my belief is founded is of this nature: 1. Bacteria and their allies are found in greatest abundance during the life of the individual in connection with dying tissue-elements, and apparently are as plentiful with dying epithelium of the cutaneous ducts, as in parts like the mouth, which are most liable to contamination with organisms from without. Again, they exist abundantly in and about the dying cells of bronchial mucus, although living bacteria appear to be almost completely absent from ordinary air.

"2. The microscopical examination of such epithelial or mucous elements also favors the notion that the contained bacteria are products engendered within such cells rather than mere results of an external contamination and imbibition. This opinion is based upon the following considerations. Bacteria only appear within the cell when it is obviously dying; and in the case of the epithelium, for instance, they manifest themselves at first as minute motionless particles scattered through the semi-solid substance of the cell, where each particle grows into a distinct bacterium, which still remains motionless, and does not appear to divide for a long time. This is precisely similar to what I have observed over and over again, when amœbæ in vegetable infusions get into an unhealthy condition and become resolved into nests of bacteria. They may exist for days in a state of activity with bacteria in the fluid around them, though none are to be seen in their interior. After a time, however, the chemical constitution of the fluid seems to become no longer suited to the amœbæ; their activity ceases, they remain as almost motionless balls of jelly, and soon multitudes of the minutest particles appear throughout their substance, each of which straightway grows into a bacterium. The former amœba is converted into a mere bag of bacteria, which after a time ruptures, and thus liberates its swarming colony of newly-engendered living units. Multitudes of mucous corpuscles seem to undergo the same kind of change, so that bacterial degeneration takes place in the same manner and is almost as typical amongst them as is fatty degeneration amongst pus-corpuscles. The two kinds of degeneration, moreover, commonly occur side by side in epithelial dèbris. Bacterial degeneration takes place where the vitality of the unity is lowered, but where it is not sufficiently degraded to permit of the still lower and more obviously distinctive process of fatty degeneration; and if any one wishes to see it in perfection let him examine some central portion of the kidney or other internal organ of a warm-blooded animal five days or more after its death."

"3. Bacteria are admitted by nearly all pathologists to be absent from the blood of healthy persons during life, and yet in from eight hours to four or five days after death, according to the temperature of the air at the time, the previously germless blood of individuals may be found to be swarming with these organisms in every stage of growth."

"4. Whereas blister fluid or serum has been shown to be free from organisms in healthy persons, I have ascertained that, given a febrile patient with a temperature of 102° F., one can determine the presence of bacteria, as will, in any blister-bleb which remains intact for forty-eight hours or more, and this, too, when the patient does not suffer from any specific fever, but merely from pneumonic inflammation. I was led to ascertain this fact by finding, about eighteen months ago, myriads of bacteria in all the blebs of a patient suffering from acute pemphigus, with a temperature of 103°."

"5. Lastly, as Dr. Sanderson has shown, a chemical irritant, such as liquor ammoniæ, may be introduced beneath the skin of some of the lower animals in such a way as to 'preclude the possibility of external contamination,' and yet here, amidst tissues which he has shown to be germless, we may thus, within twenty-four hours, determine the presence of swarms of germs and organisms in the pathological fluids effused under the influence of the local chemical irritant."

"This constitutes, as it appears to me, an exceedingly strong body of evidence tending to show that bacteria are pathological products capable of being engendered within the body after death."

The untenability of the germ-theory has been so ably demonstrated by Prof. Bastian in his address as to require no further comment. In explanation of the origin of bacteria in those diseases in which they have been met with in the tissues, he offers his own theory of "heterogenesis" and "archebiosis." In examining and considering this subject more thoroughly and without prejudice it becomes difficult to perceive any impossibility in bacteria arising, at least, by heterogenesis, as Bastian designates that process by which the particles of living tissues, on the eve of the death of the latter, may individualize themselves and continue to live in the form of a low organism. For my own part, I venture to endorse what he says.* "Just as the life of one of the cells of a higher organism may continue for some time after the death of the organism itself, so, in accordance with this latter

* *Bastian—The Modes of Origin of Lowest Organisms.* 1871, p. 15.

view, may one of the particles of such a cell be supposed to continue to live after even cell-life is impossible." That cells do live many hours after the death of the animal or man is corroborated by my own observation which I made in 1867—when examining the epithelium of the mucous membrane of the frontal sinuses of a yellow fever patient eleven hours after death—and consisting in the active motion of the cilia of a single epithelial cell, causing a movement of the cell itself, similar to that of an infusorium. The same phenomenon has been observed on ciliated epithelial cells, even at a longer time after death, by other observers. As regards Bastian's theory of archebiosis, it is more difficult to accept it at present, though the fact that there must have been a time in the history of the earth when organic matter arose from inorganic, and in its turn gave rise to the first living organisms, cannot be overcome. The theory of the development of bacteria from putrefying starch granules has been advanced previously to Bastian by *Hartig*, who has even repeated his former assertions. The origin of the minute moving granules found in the vesicles of variola and vaccina, which *Chauveau* regarded as the carriers of the specific poison, also, would find an easy and reasonable explanation by the heterogenetic theory. We may furthermore refer to the observations of Lewis on dead mice and rats, cited already in the preceding pages; and lastly to some of my own, as follows: In 1867, in examining the brains of yellow fever cases, I put small pieces of the cortex cerebri in small wide-mouthed bottles containing a solution of one-third of alcohol to two-thirds of water. The bottles were filled up to the top, so that when the cork was inserted the liquid would run over, and no air be left within. In examining these pieces four days afterward, I found great numbers of minute organisms in their interior. Some of them represented micrococcus, even zogloæ, and vibrios; but there were others, rod-like forms with highly refractive granules, unknown to me at that time, but which I found afterward explained by the labors of Koch and Ewart. In these cases, however, I shall not preclude the possibility of some of these organisms having adhered to the surface of the brain before they were put into the alcoholic solution.

[TO BE CONTINUED.]

